# Morphological Diversity and Genetic Relationships in Pulque Production Agaves in Tlaxcala, Mexico, by Means of Unsupervised Learning and Gene Sequencing Analysis

**DOI:** 10.3389/fpls.2020.524812

**Published:** 2020-09-08

**Authors:** Laura Trejo, Miguel Reyes, Daniela Cortés-Toto, Elvira Romano-Grande, Lizbeth L. Muñoz-Camacho

**Affiliations:** ^1^ Laboratorio de Biodiversidad y Cultivo de Tejidos Vegetales, Instituto de Biología, Universidad Nacional Autónoma de México, Tlaxcala, Mexico; ^2^ Departamento de Actuaría, Física y Matemáticas, Universidad de las Américas Puebla, Puebla, Mexico

**Keywords:** plant domestication, landrace, morphology, molecular markers, *Agave salmiana*, *Agave mapisaga*

## Abstract

Pulque is one of the oldest fermented beverages, with its origins dating back to pre-Hispanic Mexico. Recently, public consumption has increased. However, the majority of *Agave* plantations for pulque production have disappeared or been abandoned in recent decades. To create strategies for the conservation and production of pulque agaves, it is necessary to first determine their taxonomic identities and to better understand their genetic and morphological diversity. Despite the historical importance of pulque in Mexico, little attention has been placed on the study of *Agave* plants used for its production. Therefore, we analyzed the morphological diversity of vegetative characters of nine landraces of two *Agave* species (*A. salmiana* and *A. mapisaga*) which are widely cultivated for pulque production in Tlaxcala, Mexico. The analysis of morphological characters showed that the landraces largely clustered based on classic taxonomic relationships. One cluster of landraces associated with *Agave mapisaga* var. *mapisaga* and another with *A. salmiana* subsp. *salmiana*, but with the exception of *A. salmiana* subsp. *salmiana* “Ayoteco”, which is more closely related with *A. mapisaga* var. *mapisaga*. Additionally, we analyzed the genetic relationships between 14 landraces and wild individuals using molecular markers (*trnL* and *ITS*). The identified genetic variants or haplotypes and genetic pools mainly corresponded with the species. In the case of “Ayoteco”, incongruence between markers was observed. Low selection intensity, genetic flow events, and the plasticity of morphological traits may explain the high number of landraces without clear differences in their morphological diversity (vegetative characters) or genetic pools. The use of reproductive traits and massive sequencing might be useful for identifying possible morphological and genetic changes in the *Agave* landraces used for pulque production.

## Introduction

The *Agave* L. genus has great economic, cultural, and ecological importance in Mexico and beyond. For over 10,000 years, agaves have provided many products, such as food, fiber, construction materials, and beverages (Gentry, 1982; Colunga-GarcíaMarín et al., 2007). From an ecological perspective, agaves are the keystone species in their native environments, providing food, refuge, and water to various organisms as well as preventing soil erosion (Gentry, 1982). The genus *Agave*, commonly known as maguey, is comprised of 206 species endemic to the Americas (Gentry, 1982; García-Mendoza, 2011). It has a recent origin (7.8–10.1 mya; Good-Avila et al., 2006), with Mexico being the primary center of diversity of the genus. Nearly 75% (159) of *Agave* species are found in Mexico (Gentry, 1982; García-Mendoza, 2011). It has been suggested that *Agave* species rapidly diversified with the coinciding of increased aridization of portions of North America (Good-Avila et al., 2006) with the increased diversity of pollinator species, which favored hybridization of *Agave* species (Good-Avila et al., 2006; Flores-Abreu et al., 2019).

One of the most important products obtained from *Agave* plants is pulque, a fermented beverage of pre-Hispanic origin. However, little to no research has been carried out on the *Agave* species used for pulque production, making it difficult to develop adequate conservation, management, and production strategies. Pulque agaves (*magueyes pulqueros*) are enormous plants that can measure more than 5 m in diameter and 10 m in height, including their inflorescence. These plants are mainly adapted to the elevation and climatic conditions of central Mexico. The main species utilized in pulque production are *Agave* *americana* L., *Agave inaequidens* Koch, *Agave mapisaga* Trel., *Agave salmiana* Otto ex Salm-Dyck subsp. *salmiana*, and *Agave salmiana* Otto ex Salm-Dyck subsp. *tehuacanensis* (Karw. Ex Salm-Dyck) García-Mend. (García-Mendoza, 2011). One study found more than 60 common names for pulque agaves (Mora-López et al., 2011), complicating the study of their diversity.

Pulque is produced by the alcoholic fermentation of *Agave* sap, which is called aguamiel. Its consumption, along with its benefits, has been recorded in at least eight codices since pre-Hispanic times (Gonςalves de Lima, 1956). According to the Aubin Codex, the Aztecs discovered pulque in the year 7 Acatl (1187 A.D.) during their long pilgrimage from Aztlán in search of Tenochtitlan (which is now Mexico City; Gonςalves de Lima, 1956). During the pre-Hispanic period, pulque played a major role in the cultural development of the region, especially for populations living in the Mexican highlands. For example, pulque was offered as tribute to the Aztecs by those they conquered (Guerrero-Guerrero, 1985). However, on a regular basis, this beverage was supposedly only consumed by the elderly, the sick, lactating women, and high-level dignitaries. Being inebriated by pulque was considered improper and a cause for punishment (Gonςalves de Lima, 1956). Alternatively, the Florentine Codex states that pulque was sold in the so-called *tianguis*, or open-air markets, and was consumed by those who participated in religious ceremonies as well as manual laborers (Sahagún, 1979).

Such was the economic importance of pulque that its production was allowed to continue in New Spain despite the prohibition of many other native beverages, such as, for example, mezcal. At the time of independence, 30,000 barrels of pulque were being produced every year (Guerrero-Guerrero, 1985). By the beginning of the Mexican Revolution in 1910, however, agrarian reform led to a 45% reduction in production which was also a result of land redistribution, *Agave* overexploitation, competition from other alcoholic beverages (mainly beer), and an intense smear campaign (Guerrero-Guerrero, 1985; Ramírez-Rancaño, 2000). From that point, pulque was considered a filthy, unsanitary, and low-class beverage dangerous for human health (Ramírez-Rancaño, 2000). Given the decrease in consumption, *Agave* plants have gradually disappeared from the Mexican landscape, with the majority of production fields being abandoned or substituted by other crops (Ramírez-Rancaño, 2000; Alvarez-Duarte et al., 2018).

Despite these historical challenges, pulque has had a rebound in recent years due to the efforts of the Mexican government to promote it through fairs, its consumption among young people and also due to the existence of economic resources to promote the cultivation of agaves used to produce it (Ramírez, 2014). Various studies have shown that pulque is, in fact, safe for consumption and nutritious, with high levels of carbohydrates, mineral salts, vitamins, and amino acids (Escalante et al., 2008; Lappe-Oliveras et al., 2008; Ortiz-Basurto et al., 2008). It also contains prebiotic and probiotic microorganisms that help improve intestinal microbiota (Moreno-Vilet et al., 2014). Many other products are obtained from pulque agaves including: inulin-type fructans; xylitol sweetener (Alvarado et al., 2014); agave syrup; distilled liquors, such as mezcal; leaves for cooking barbacoa (Gentry, 1982; Colunga-GarcíaMarín et al., 2007); *mixiotes*, or leaf cuticles for wrapping and cooking meat; the edible white worm (*Aegiale hesperians* Walker) and red worm (*Hipota agavis* Blásquez); and maguey mushrooms (*Pleurotus opuntiae* Durieu et Levy; Gentry, 1982; Alvarez-Duarte et al., 2018).

Given the context provided above, the present study seeks to contribute to the understanding of the morphological and genetic diversity of traditional pulque *Agave* species and landraces in Tlaxcala, the second-most important state in Mexico in terms of pulque production (Álvarez-Duarte et al., 2018; Ramírez-Manzano, 2020), through an analysis of morphological characters and chloroplast and nuclear DNA markers. The resulting data are fundamental for the conservation of the landrace diversity of pulque *Agave* species and for improving their production in central Mexico.

## Materials and Methods

### Study Area

The state of Tlaxcala is located in the central eastern region of Mexico between 19° 06’ 18’’ and 19° 43’ 44’’ N and 97° 37’ 31’’ and 98° 42’ 30’’ W, with an elevation range of 2200 to 4,400 m above sea level. It comprises 0.2% of the area of Mexico (399,113.7 ha) and has a population of 1,272,847. Its climate can be characterized as semi-arid and cool temperate, with an annual precipitation of 720 mm (INEGI, 2016). The main soil type is cambisol. Agriculture is one of the main economic activities and corresponds with 74% of the state’s area; the main crops are corn (*Zea mays* subsp. *mays* L.), beans (*Phaseolus vulgaris* L.), barley (*Hordeum vulgare* L.), and potato (*Solanum tuberosum* L.). Nearly 41% of the land area is comprised of grasslands, and another 9% of forests, wherein the main arboreal species are *Pinus teocote* Schiede ex Schltdl., *Juniperus depeana* Steud., *Abies religiosa* (Kunth) Schltdl & Cham., and *Quercus laurina* Humb & Bonpl (INEGI, 2016).

### Study System

**Figure 1:**
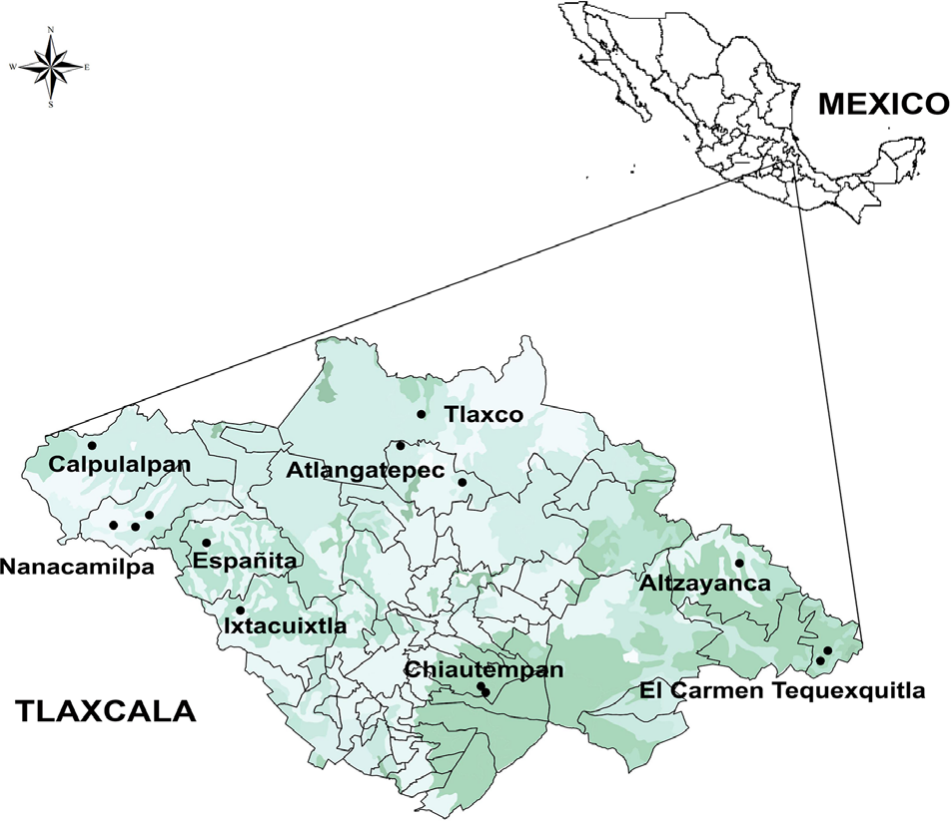
Map of localities (circles) where measurements on plants cultivated in ranches, plots, and milpas (mixed crop plots) were carried out. This map presents a land use and vegetation layer (Qgis 2.6.1-Brighton).

Fourteen *Agave* landraces used for pulque production in Tlaxcala and three wild individuals (plants from wild populations that are not used for pulque production) were analyzed (**Table 1** and **Table S1**). Landraces are plants that are only grown in certain ecogeographic areas that have adapted to the environmental conditions of these areas as well as traditional management practices and uses (Casañas et al., 2017). Both landraces and wild individuals were identified at the species and subspecies level (**Table 1** and **Table S1**) following García-Mendoza’s (2011) and Gentry’s (1982) taxonomic keys. However, there are no taxonomic keys specifically for identifying landraces, which are generally identified by their common names (names assigned by people). These landraces are described in the present study according to our field work observations in nine municipalities, 10 localities, and 14 producers (see **Supplementary Data 1**, **Figure 1**).

**Table 1:** Morphological diversity of nine *Agave* landraces analyzed, species, places of origin and number of individuals by population.

Species	“Landrace”	Municipality	Community	Individuals
*A. salmiana* subsp. *salmiana*	“Amarillo”	Chiautempan	San Pedro Tlalcuapan	14
*A. salmiana* subsp. *salmiana*	“Ayoteco”	Nanacamilpa	Nanacamilpa	27
*A. salmiana* subsp. *salmiana*	“Ayoteco”	Nanacamilpa	Nanacamilpa	25
*A. salmiana* subsp. *salmiana*	“Colorado”	Atlangatepec	Villa Alta	10
*A. salmiana* subsp. *salmiana*	“Colorado”	Nanacamilpa	Nanacamilpa	10
*A. salmiana* subsp. *salmiana*	“Chalqueño”	Nanacamilpa	Nanacamilpa	24
*A. salmiana* subsp. *salmiana*	“Chino”	Ixtlacuixtla	Alpotzonga	25
*A. salmiana* subsp. *salmiana*	“Manso”	Altzayanca	Altzayanca	26
*A. salmiana* subsp. *salmiana*	“Manso”	Atlangatepec	Villa Alta	21
*A. salmiana* subsp. *salmiana*	“Manso”	Calpulalpan	Xultepec	25
*A. salmiana* subsp. *salmiana*	“Manso”	Chiautempan	San Pedro Tlacoapan	25
*A. salmiana* subsp. *salmiana*	“Manso”	El Carmen Tequexquitla	El Carmen Tequexquitla	18
*A. salmiana* subsp. *salmiana*	“Manso”	El Carmen Tequexquitla	El Carmen Tequexquitla	17
*A. salmiana* subsp. *salmiana*	“Manso”	Ixtlacuixtla	Alpotzonga	25
*A. salmiana* subsp. *salmiana*	“Manso”	Nanacamilpa	Nanacamilpa	34
*A. salmiana* subsp. *salmiana*	“Manso”	Nanacamilpa	Nanacamilpa	35
*A. salmiana* subsp. *salmiana*	“Manso”	Nanacamilpa	Nanacamilpa	19
*A. salmiana* subsp. *salmiana*	“Manso”	Tlaxco	Hacienda Xochuca	25
*A. salmiana* subsp. *salmiana*	“Prieto”	Chiautempan	San Pedro Tlalcuapan	25
*A. salmiana* subsp. *salmiana*	“Xilomelt”	Españita	Álvaro Obregón	13
*Agave mapisaga* var. *mapisaga*	“Palmilla”	Atlangatepec	San Pedro Ecatepec	13
*Agave mapisaga* var. *mapisaga*	“Palmilla”	Españita	Álvaro Obregón	22

### Morphological Diversity

**Figure 2:**
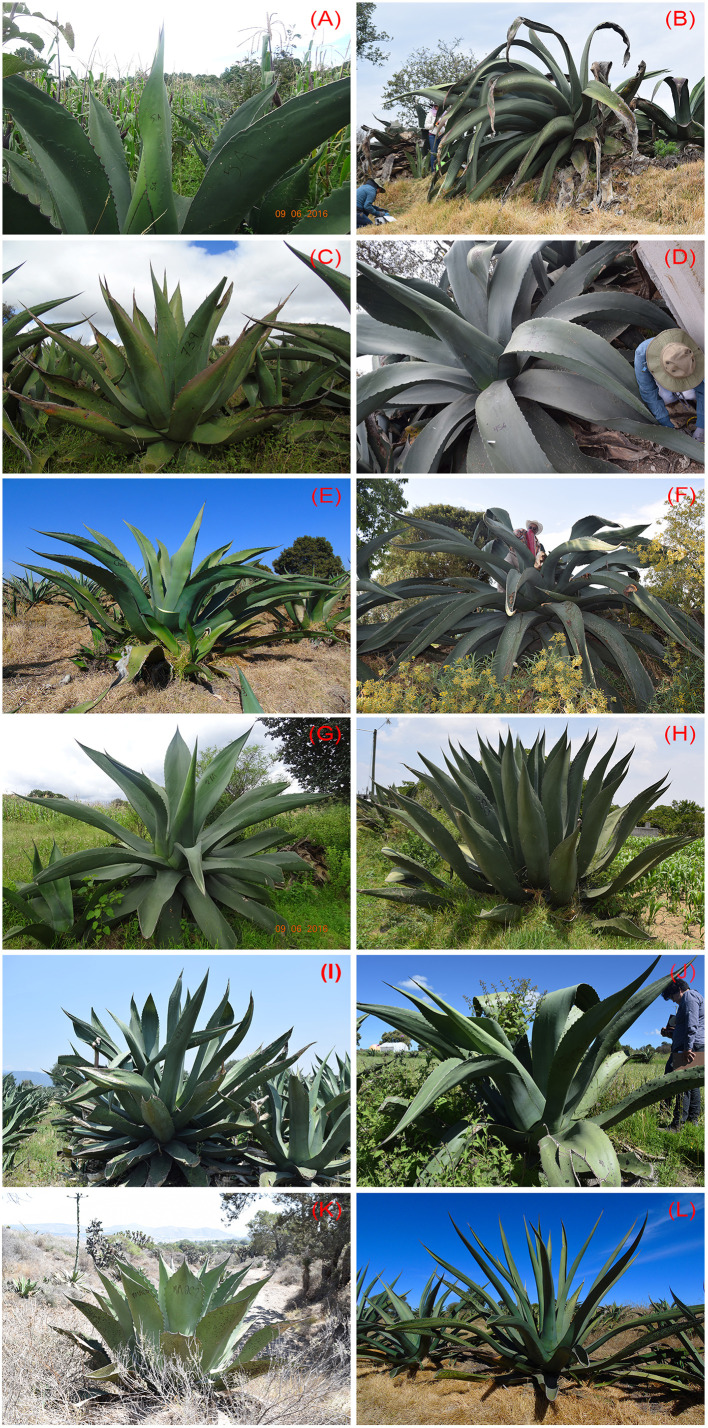
Agaves used in the production of pulque. **(A–J)** *Agave salmiana* subsp. *salmiana*: **(A)** “Amarillo,” **(B)** “Ayoteco,” **(C)** “Colorado,” **(D)** “Chalqueño,” **(E)** “Chino,” **(F)** “Manso,” **(G)** “Matecón,” **(H)** “Prieto,” **(I)** “Púa Larga,” **(J)** “Xilomelt.” **(K)** *Agave salmiana* subs *tehuacanensi*s Tepezorra and **(L)** *Agave mapisaga* var. *mapisaga* “Palmilla.”

Nine *Agave* landraces were analyzed (**Figure 2**): *Agave salmiana* subsp. *salmiana* “Amarillo”, “Ayoteco”, “Colorado”, “Chalqueño”, “Chino”, “Manso”, “Prieto”, and “Xilomelt” and *Agave mapisaga* var. *mapisaga* “Palmilla”. Twenty-two populations of 13–35 individuals were included in the study (N = 478 individuals; **Table 1**; https://zenodo.org/record/3976297#.Xy35gChKjIU). The number of landraces and analyzed individuals depended on the availability of the number of mature *Agave* plants close to initiating inflorescence emergence.

**Figure 3:**
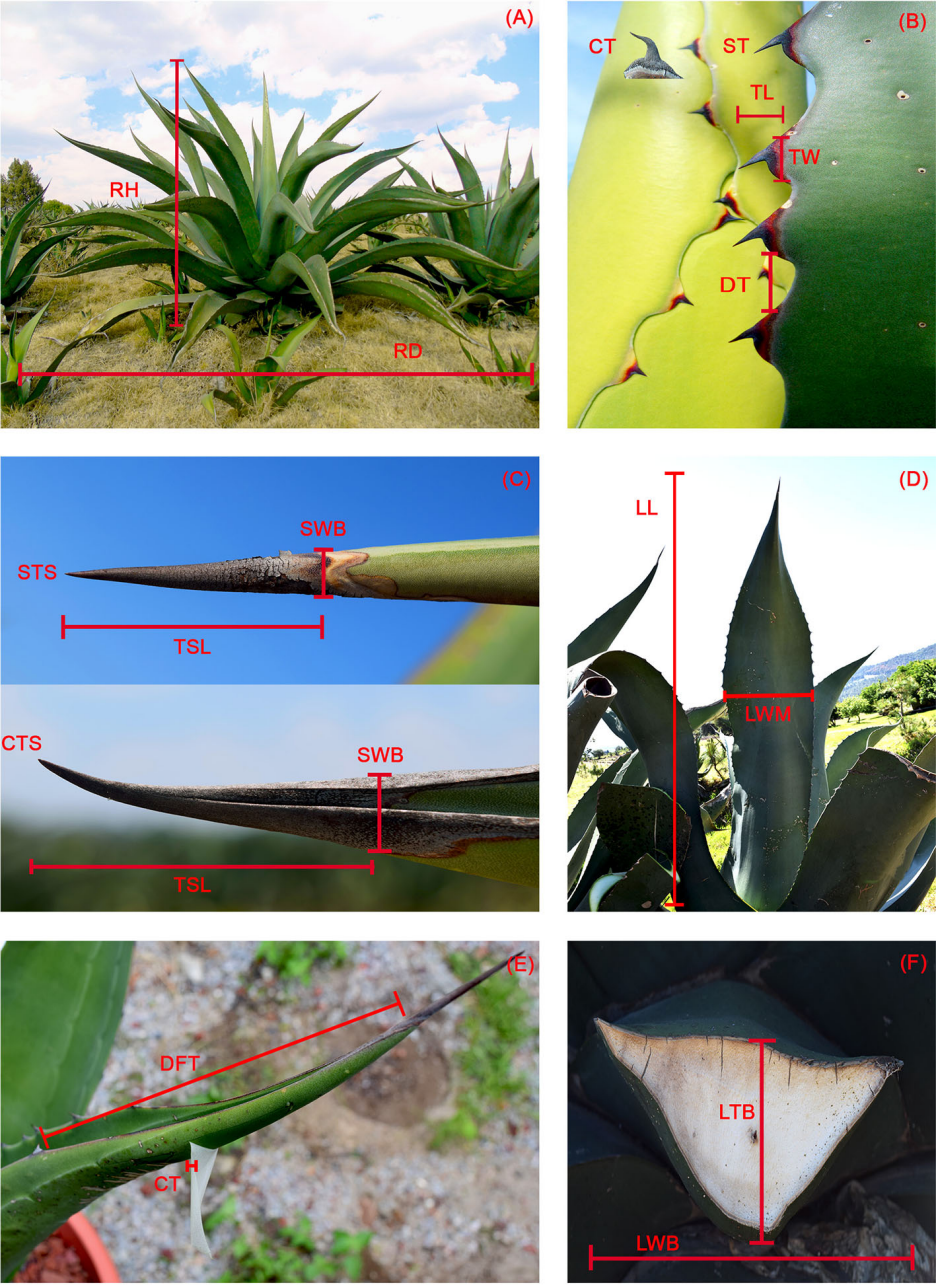
Morphological characters. **(A)** RH, rosette height; RD, rosette diameter. **(B)** CT, curved teeth; ST, straight teeth; TL, tooth length; TW, tooth width; DT, distance between teeth. **(C)** STS, straight terminal spine; CTS, curved terminal spine; SWB, terminal spine, width at base; TSL, terminal spine length. **(D)** LL, leaf length; LWM, leaf, width at mid length. **(E)** DFT, distance to first tooth; CT, cuticle thickness. **(F)** LTB, leaf thickness at base; LWB, Leaf width at base.

Twenty morphological variables were measured (**Figure 3**) in each individual plant. Of these variables, spine shape and tooth shape were binary (0, 1), and total number of leaves, number of rigid leaves, and number of flaccid leaves were counts. The remaining variables were intervals except for the percentage of rigid leaves, which was treated as an ordinal variable with five levels since most leaves were rigid (i.e., 77% of observations had entirely rigid leaves and 82% of the observations had at least 95% rigid leaves). It was calculated from three variables (total number of leaves, number of rigid leaves, number of flaccid leaves). The levels were as follows: 1) less than 60% rigid leaves, 2) 60 to 70% rigid leaves, 3) 70 to 80% rigid leaves, 4) 80 to 90% rigid leaves, and 5) more than 90% rigid leaves.

**Figure 4:**
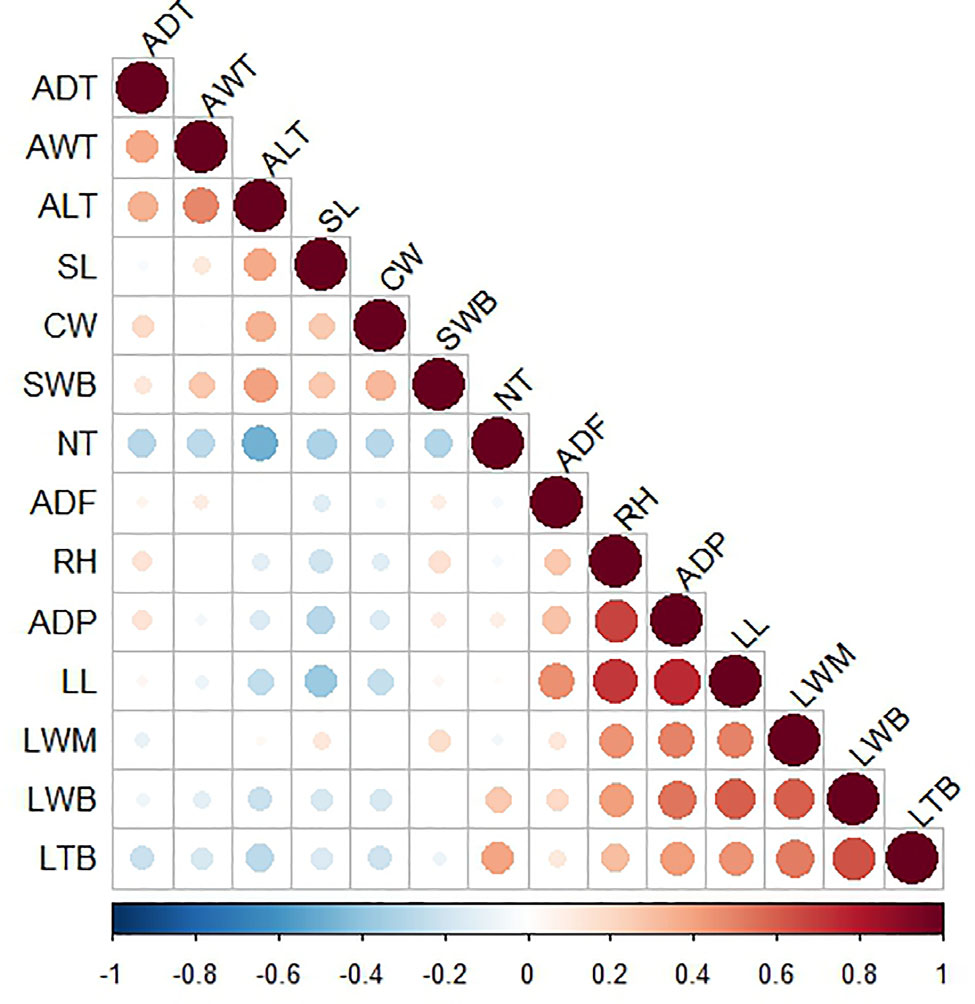
Heat map of linear correlations between interval variables. Intense red corresponds with a highly positive correlation and intense blue with a highly negative correlation. Size of the circles indicate the magnitude of the correlation coefficient: larger circles indicate greater correlation, being the maximum size (in the diagonal) equivalent to a coefficient of 1. RH, Rosette height (cm); ADP, Average diameter of plant (cm); TNL, Total number of leaves; NRL,Number of rigid leaves; NFL, Number of flaccid leaves; CW, Cuticle width (mm), LL, Leaf length (cm); LWB, Leaf width at base (cm); LWN, Leaf width at middle (cm); LTB, Leaf thickness at base (cm); SL, Spine length (mm); SWB, Spine width at base (mm); SS, Spine shape; ADF, Average distance of the first tooth (cm); NT, Number of teeth; ADT, Average distance between teeth (mm); AWT, Average width of teeth (mm); ALT, Average length of teeth (mm); TS, Tooth shape; PRL, Percentage of rigid leaves.

Pearson linear correlations were calculated between interval variables to detect redundant information and, in this way, describe the morphology of the plants with a smaller number of variables. The correlations are shown on the heat map in **Figure 4**. Variables with correlations above *R* = 0.11 appear collapsed in the same group, all of them providing redundant information. Based on correlations and a PCA (Gabriel, 1971; Gabriel, 1980), we selected three variables (average distance of the first tooth, leaf length, and leaf thickness at the base) from the first group in addition to three variables (average length of teeth, cuticle width, and spine length) from the second group (see **Figure 4** and **Figure S1**). We also included two binary variables (spine shape and tooth shape) and the ordinal variable (percentage of rigid leaves). Consequently, we were able to describe the morphology of the plants with just nine variables (i.e., the morphology of each individual plant is represented by a 9-dimensional vector) (Nguyen and Holmes, 2019).

In order to generate clusters of plants based on morphological data, we used a hierarchical agglomerative clustering algorithm (Rokach and Maimon, 2005) along with the Gower’s metric (Gower, 1971), which is suitable for calculating distances between mixed data (i.e., individuals described using different types of variables, such as binary, ordinal, and interval; Kaufman and Rousseeuw, 2005). We calculated these distances between our mixed data using the daisy function in the cluster package (Maechler et al., 2018) of R software (R Core Team, 2019). Once the dissimilarity matrix was obtained, we used the hierarchical clustering algorithm in the agnes function of the aforementioned package. For the linkage criterion, we used the Ward’s method (Ward, 1963).

The visualization of the clustering structure is useful for deciding the number of clusters. For this, we used a dendrogram, which is a graphical tool that shows the distance at which observations merged. We also used the agglomerative coefficient, which describes the strength of the obtained clustering structure, ranging from zero to one. Values close to one suggest a strong clustering structure, and values close to zero a weak structure (Kaufman and Rousseeuw, 2005). Both the dendrogram and agglomerative coefficient can be obtained *via* the plot fuction applied to a proper agnes object.

For a visual impression of the detected clusters, we used an Andrews plot. High-dimensional data can be represented defining a finite Fourier series, so that each multivariate vector is represented by a curve in a two-dimensional space. Due to the periodicity of Fourier series, the *x*-axis is the interval from −π to π (roughly from −3.14 to 3.14), while the *y*-axis is the value of the specific finite Fourier series (defined in each situation) when evaluated over the interval from –π to π (Andrews, 1972). If there is structure in the data, it should be visible in the Andrews curves.

The curve algorithm was implemented using the Andrews function in the Andrews package in R software (Myslivec, 2012).

### Genetic Relationships

Twenty individuals of *A. salmiana* subsp. *salmiana*, *A. salmiana* subsp. *tehuacanensis*, and *A. mapisaga* var. *mapisaga* were included in the relationship analysis. Seventeen belonged to the landraces, and three were wild types (*A. salmiana* subsp. *salmiana* Manso Listado, *A. salmiana* subsp. *salmiana* Prieto Silvestre, and *A. salmiana* subsp. *tehuacanensis* Tepezorra; **Table S1**). Tissue from the tip of a leaf was collected from each individual and kept in silica gel. DNA was extracted from each sample using liquid nitrogen and the DNeasy Plant Mini Kit extraction kit (Qiagen, Hilden, Germany). The quantity and quality of the obtained DNA were measured using a Colibri microvolume spectrometer (Titertek-Berthold, Pforzheim, Germany).

Ten primers previously used to screen for interspecific variation in *Agave* were tested (Good-Avila et al., 2006; Scheinvar et al., 2017; Flores-Abreu et al., 2019). Only two of them presented variation (**Table S2**): the *trnL* ^(UAA)^ intron (c–d) of cpDNA (Taberlet et al., 1991) and the internal transcribed spacers (*ITS*) 1–4 (Bayer et al., 1996). The *trnL* ^(UAA)^ primer was amplified by polymerase chain reaction (PCR) under the following protocol: 95°C/7 min and 35 cycles of 95°C/1 min, 52.4°C/60 s, and 72°C/90 s followed by one cycle of 72°C/7 min and one cycle of 4°C/10 min. The nuclear ribosomal DNA *ITS* 1–4 were sequenced under the following protocol: 94°C/2 min followed by 30 cycles of 94°C/1 min, 57°C/90 s, 72°C/2 min; an elongation period of 72°C/10 min; and one cycle of 4°C/10 min. The PCR was performed for both pairs of primers, each reaction had a final volume of 25 µL and contained: 2 µL of DNA (10–100 ng), 1 µL of 10 µM for each pair of primer, and 21 µL (1X) of GoTaq Hot Start Colorless Master Mix (Promega, Madison, USA). The PCR products were run in an electrophoresis chamber in agarose gel and purified with QIAquick PCR purification kits (Qiagen). The sequences were obtained in the Biodiversity and Health Genomic Sequencing Laboratory of the Institute of Biology, Universidad Nacional Autónoma de México (UNAM). To prepare the samples, we used 0.4 µL of big dye terminator v. 3.1, 2 µL of 5x buffer, 4 µL of water, 1 µL of primer (10 µM), and 3 µL of the purified amplificate. The samples were placed in the PCR, and the following program was used: 30 cycles at 96°C/10 s, 50°C/5 s, and 60°C/4 min. Once the cycle series ended, the samples were purified with Centri-Sep (Thermo Fisher Scientific) following the manufacturer’s specifications. To the purified samples, 25 µL of EDTA (0.5 mM) were added, and the samples on the plate were run in an Applied Biosystems 3730xL sequencer (ThermoFisher Scientific). The generated sequences were uploaded to GenBank (see **Table S1**).

Sequences were cleaned and aligned by eye with the support of BioEdit 7.2.6.1. Haplotype networks per marker and per combined matrix were obtained, and a parsimony analysis was performed using the TCS 1.21 program (Clement et al., 2000). The gaps were considered as missing data, and the network was constructed at a 95% confidence level.

For each marker and combined matrix, a Bayesian clustering method was applied with the support of the STRUCTURE v. 2.3.4 program. For these analyses, an independent allelic frequencies linkage model was used (Pritchard et al., 2000; Falush et al., 2003). A total of 350,000 Markov Chain Monte Carlo (MCMC) repetitions were performed, with 150,000 burn-in periods for each run. Ten runs were designed to estimate from 1 to the 5 populations. The optimum number (K) of genetic clusters was obtained by calculating the ΔK statistic using an Evanno test in the STRUCTURE HARVESTER program (Evanno et al., 2005; Earl and vonHoldt, 2012). A bar plot was made to represent the individual assignment probability obtained for K in the R software.

## Results and Discussion

### Morphological Diversity

In the different analyses carried out under the unsupervised learning method, we mainly observed two clusters related with each species. All of the landraces of *A. salmiana* subsp. *salmiana*, with the exception of “Ayoteco”, were mainly observed in one of the clusters, and the other cluster was mainly composed of *A. mapisaga* var. *mapisaga* and *A. salmiana* subsp. *salmiana* “Ayoteco”. This confirms that the analysis of only the vegetative characters can distinguish the *Agave* species but not landraces of commonly used for pulque production. However, the landrace “Ayoteco” recognized in classical taxonomy as *A. salmiana* subsp. *salmiana* is closer to *A. mapisaga* var. *mapisaga* based on morphometric evidence, as provided below.

**Figure 5:**
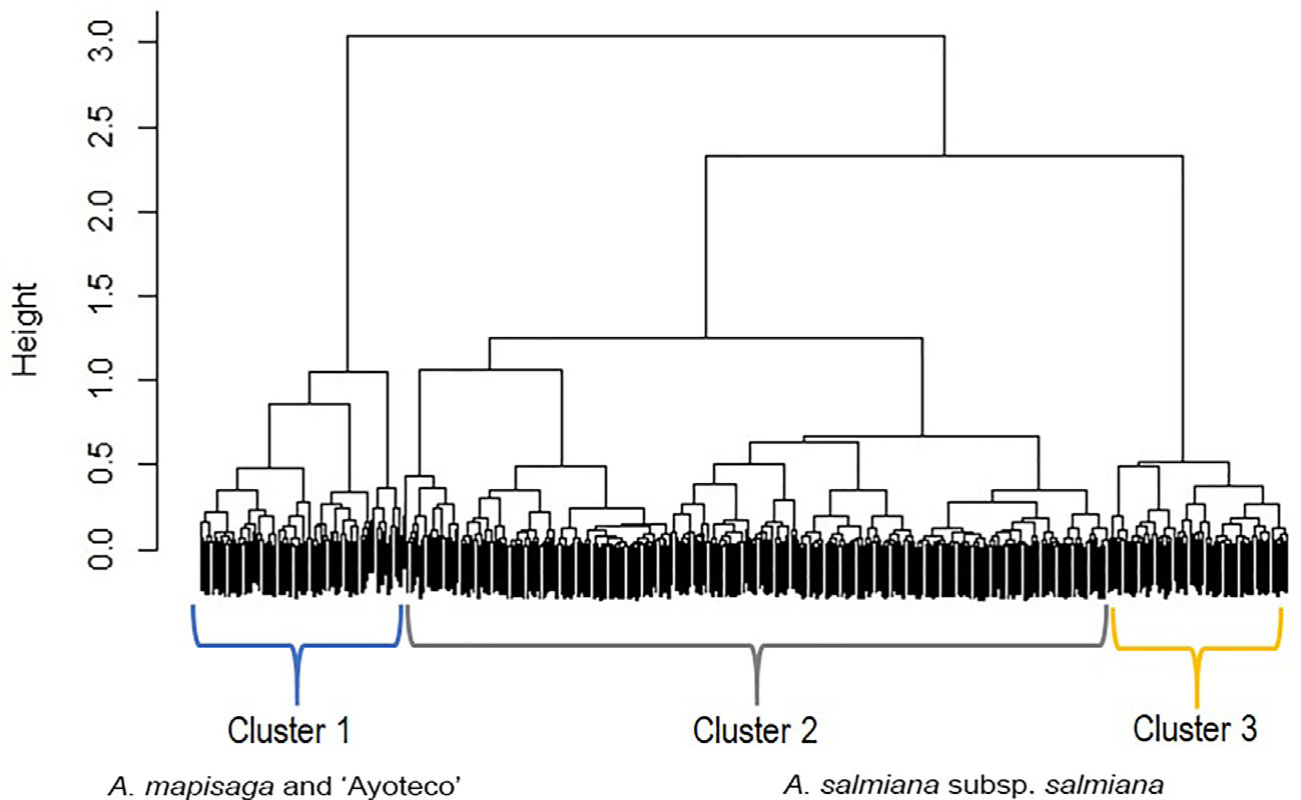
Dendrogram showing the fusions of individual observations when clustering according to the “agnes” function of the R cluster library using Gower’s dissimilarities and Ward’s method. Agglomerative coefficient: 0.9855. The black area on the bottom represents the unclustered data, while the black vertical lines that protrude into the white area show the heights (dimensionless distance) at which the clusters were formed.

**Figure 6:**
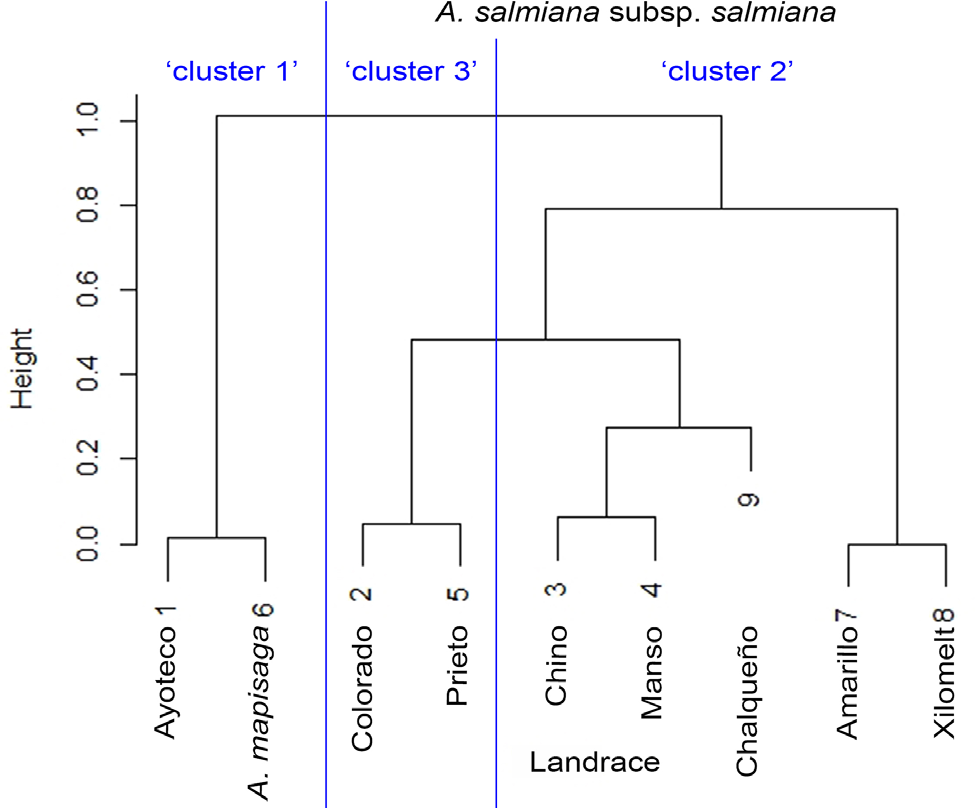
Similarity dendrogram between varieties. The percentage distributions in **Table 2** were introduced as vectors in the “agnes” function of the R cluster library using Ward’s method.

The dendrogram (**Figure 5**) is overall divided into two large morphological zones: a first zone indicated by cluster 1 and a second zone indicated by merger of cluster 2 and cluster 3. However, a further split of the merged cluster 2 and 3 is reasonable as well. Clusters 2 and 3 consisted of landraces of *A. salmiana* subsp. *salmiana*, with the exception of “Ayoteco”, and cluster 1 contains mainly *A. mapisaga* var. *mapisaga* and *A. salmiana* subsp. *salmiana* “Ayoteco”. **Table 2** shows how the individuals of the landraces are distributed in the three clusters as well as the total observations in each. To formalize the distribution of the individual plants according to landrace and cluster, we performed a cluster analysis using the same agglomerative method and the Ward’s method with the row-wise vectors in **Table 2B** (**Figure 6**).

**Table 2:** **(A)** Distribution of the different individual plants according to landrace and cluster, in absolute numbers. **(B)** Proportions of individual plants distributed (row wise) according to landrace and cluster.

Landrace	(A)				(B)			
	Absolutes				Proportions			
	Cluster 1	Cluster 2	Cluster 3	Total	Cluster 1	Cluster 2	Cluster 3	Total
*A. mapisaga* var. *mapisaga* “Amarillo”	0	**14**	0	14	0.00	**1.00**	0.00	1.00
*A. salmiana* subsp. *salmiana* “Ayoteco”	**29**	23	0	52	**0.56**	0.44	0.00	1.00
*A. salmiana* subsp. *salmiana* “Colorado”	2	**10**	8	20	0.10	**0.50**	0.40	1.00
*A. mapisaga* var. *mapisaga* “Chalqueño”	7	**16**	1	24	0.29	**0.67**	0.04	1.00
*A. salmiana* subsp. *salmiana* “Chino”	3	**18**	4	25	0.12	**0.72**	0.16	1.00
*A. salmiana* subsp. *salmiana* “Manso”	25	**188**	57	270	0.09	**0.70**	0.21	1.00
*A. salmiana* subsp. *salmiana* “Prieto”	3	**13**	9	25	0.12	**0.52**	0.36	1.00
*A. salmiana* subsp. *salmiana* “Xilomelt”	0	**13**	0	13	0.00	**1.00**	0.00	1.00
*A. mapisaga* var. *mapisaga* “Palmilla”	**20**	15	0	35	**0.57**	0.43	0	1.00
Total	89	310	79	478				

The highest values for each landrace were shown in bold.

**Table 3** shows the means of the interval variables per cluster. The main morphological contrasts occurred between individuals in cluster 1 and the merged cluster 2 and cluster 3 (**Table 3**). The landraces of cluster 1 (mainly *A. mapisaga* var. *mapisaga* and *A. salmiana* subsp. *salmiana* “Ayoteco”) had, on average, shorter average teeth length, shorter spine length, and thinner cuticles; they also have a longer distance to the first tooth, greater leaf thickness at the base, and longer leaves. On the other hand, the means of the individuals in cluster 2 and 3 (mainly landraces of *A. salmiana* subsp. *salmiana*, with the exception of “Ayoteco”) showed some differences yet tended to be more similar.

**Table 3:** Averages of the morphological interval variables per cluster.

	Cuticle width *(mm)*	Leaf length *(cm)*	Leaf thickness at base *(cm)*	Spine length *(mm)*	Average distance of the first tooth *(cm)*	Average length of teeth *(mm)*
**Cluster 1**						
Mean	0.09	226.4	20.4	49.1	24.4	4.9
Standard deviation	0.02	38.6	3.9	13.7	7.2	1.8
**Cluster 2**						
Mean	0.11	196.6	18.7	61.0	20.8	6.0
Standard deviation	0.02	36.5	4.4	11.9	6.5	2.2
**Cluster 3**						
Mean	0.10	184.1	16.9	66.4	20.7	7.3
Standard deviation	0.01	33.4	5.0	11.9	6.1	2.7

Individuals in cluster 1 also differed from those in cluster 2 and 3 in terms of spine and tooth shape (**Table 4**). Cluster 1 (mainly *A. mapisaga* var. *mapisaga* and *A. salmiana* subsp. *salmiana* “Ayoteco”) is dominated by individuals with curved spines and straight teeth. Cluster 2 (mainly *A. salmiana* subsp. *salmiana* “Amarillo”, “Chalqueño”, “Chino”, “Manso”, and “Xilomelt”) is basically formed by individuals with straight spines and straight teeth. Meanwhile, in cluster 3 (mainly *A. salmiana* subsp. *salmiana* “Colorado” and “Prieto”), all individuals have straight spines and curved teeth. Morphological differences between clusters are also evident in the percentage of rigid leaves. Ninety-five percent of individuals in cluster 3 had at least 90% rigid leaves, but only 66% of individuals in cluster 1 had rigid leaves. Meanwhile, cluster 2 is between clusters 1 and 3 in terms of percentage of rigid leaves (see **Table 5**). Therefore, it was possible to morphologically separate species of pulque agaves.

**Table 4:** Percentage of individual plants with straight spine, curve spine, straight tooth and curve tooth per cluster.

Cluster	Straight spine	Curve spine	Straight tooth	Curve tooth
Cluster 1	0	100	84	12
Cluster 2	100	0	99	1
Cluster 3	100	0	0	100

**Table 5:** Percentage distribution of the ordinal variable (percentage of rigid leaves) per cluster.

Percentage of rigid leaves per cluster	*1 (≤60%)*	*2 (>60% and ≤70%)*	*3 (>70% and ≤80%)*	*4 (>80% and ≤90%)*	*5 (>90%)*
Cluster 1	6	3	15	10	66
Cluster 2	1	3	3	6	87
Cluster 3	0	0	0	5	95

**Figure 7:**
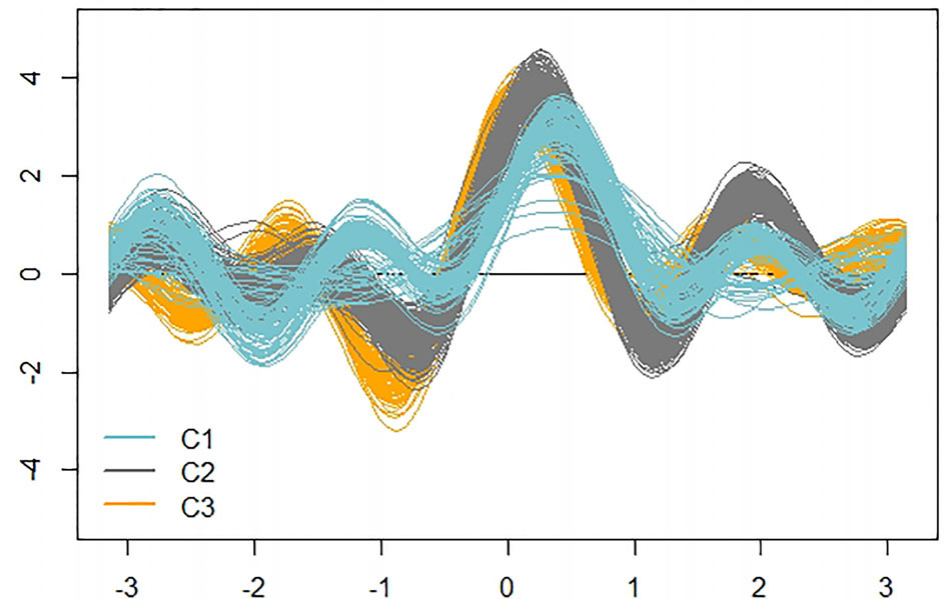
Andrews curves of the 478 observations distinguished according to cluster. Blue: cluster 1; Gray: cluster 2; Orange: cluster 3.

**Figure 7** shows the actual clusters in two dimensions. It is important to remember that the morphological vectors of the individual plants are 9-dimensional, so we cannot visualize them precisely. To have a visual impression of both the data and clusters, it is necessary to use visualization techniques such as the Andrews plot, whose main, and perhaps only, purpose is to visualize high dimensional data. This plot allowed us to confirm that the clusters are not only geometrically but visually differentiable and, as already anticipated in the dendrogram, clusters 2 and 3 are more similar, as can be seen in the greater similarity of the oscillatory patterns of the observations of these clusters.

The majority of the studies on the morphological diversity of agaves for pulque production are based solely on the analysis of vegetative characters (Alfaro-Rojas et al., 2007; Mora-López et al., 2011; Figueredo et al., 2014; Alvarez-Ríos et al., 2020; **Table S3**). Similar to the present study, the variability of morphological vegetative characters in these latter studies is distributed and grouped at the species level with respect to classical taxonomy (Mora-López et al., 2011; Alvarez-Ríos et al., 2020; **Table S4**). Mora-López et al. (2011) observed that *A. salmiana* and *A. mapisaga* var. *mapisaga* are separated into two groups, similar to the findings herein. Alfaro-Rojas et al. (2007) analyzed six stem characters and found that “Manso”, “Ayoteco”, and “Verde” formed a single cluster that differed from “Xilomelt”. Notably, these latter authors also found that “Ayoteco” belonged to the morphological group of *A. salmiana* in contrast with our findings that it is closest to *A. mapisaga* var. *mapisaga*. This reflects the difficulty of identifying plants by only their common names, as it is possible that the “Ayoteco” studied by Alfaro-Rojas et al. (2007) is not the same that we analyzed herein or that this cultivar presents variability in vegetative characters throughout its distribution.

The analysis of reproductive, biochemical, and genetic characters could improve the taxonomic resolution of the different landraces. Figueredo et al. (2014, 2017) analyzed vegetative, reproductive, and genetic characters, distinguishing wild populations of *A. inaequides* from cultivated ones as well as species such as *A. hookeri* and *A. cupreata*. Few studies have analyzed reproductive characters due to the difficulty of obtaining flowers, fruits, and seeds from pulque agaves. Growers halt their development to obtain aguamiel or, if flowers bloom, people collect them as a food source (**Tables S3** and **S4**).

Comparison between previous studies and ours is not an easy task because of differences between studies. For example, each study measured morphological characters in different ways, and the most significant characters also tended to be different in each study (**Tables S3** and **S4**). The number of measured plants has varied from 2 to 100; however, in most of cases, just a few plants were measured. In addition, many authors did not identify species and landraces using classical taxonomic keys. Rather, the majority of studies only reported the common name of the taxa, which complicates comparisons given that these names may or may not correspond with the same taxa across studies. These issues could be resolved by standardizing the methods for measuring morphological, biochemical, and genetic characters. In addition, studies should be rigorous in their taxonomic identification efforts and aspire to create taxonomic keys for identifying landraces in the future in order to avoid the ambiguity caused by using only common names.

Some authors have also pointed out that the management and artificial selection of certain plants could have modified certain characters, reflecting the characteristics desired by growers (Vargas-Ponce et al., 2007; Mora-López et al., 2011). Growers tend to select for larger plants (longer leaves and thicker leaf bases), smaller teeth, greater distance between teeth, greater distance from the apical spine to the first tooth, and shorter apical spines. Cluster 1 (“Ayoteco” and *A. mapisaga* var. *mapisaga*) could be expressing these characters of interest. Notably, we also observed that the subspecies *salmiana*, in particular the “Manso” landrace, is still cultivated in a very traditional manner in Tlaxcala. On the other hand, *A. mapisaga* and “Ayoteco” could be the landraces most modified by growers. However, these differences could also be due to pre-existing differences between species resulting from their evolutionary histories. The shape of the spine and teeth could be explained by the latter, as these do not appear to be characters of interest but rather distinctive characters of the landraces (**Table 4**). To better understand the changes caused by artificial selection, it would be necessary to make comparisons with wild relatives.

The analysis of morphological diversity has many practical applications for the management, production, and conservation of the *Agave* landraces used for pulque production. Through these studies, the variability of morphological characters in production fields can be described, possibly enabling the identification of different landraces as morphological groups with particular characters, which has taxonomic implications. The characterization of this diversity is fundamental for the establishment of *in situ* and *ex situ* conservation strategies. On the other hand, the lack or loss of diversity can lead to an increase in the vulnerability of plants to pests and diseases and decrease their resistance to climate change.

### Genetic Relationships

The low resolution obtained from the *Agave* genome fragments is most likely due to the recent origin of the genus, around 6–10 million years ago, and the high hybridization rates occurring as a consequence of pollinator-mediated genetic flow (Good-Avila et al., 2006; Scheinvar et al., 2017; Flores-Abreu et al., 2019). In our first attempt to use genetic markers to distinguish pulque-producing agaves, we only found enough variation to differentiate between species using one plastid marker and one nuclear marker.

**Figure 8:**
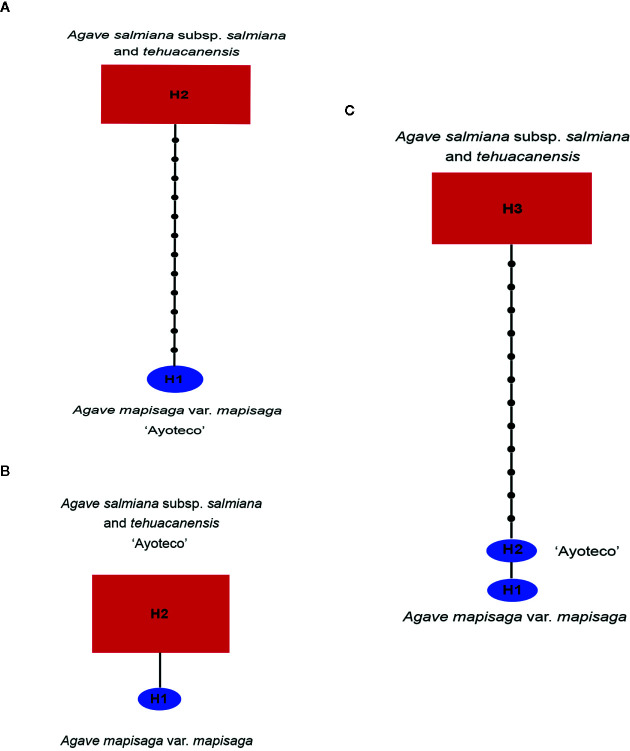
Haplotype network from the **(A)** *trnL*, **(B)** *ITS*, and **(C)** combined *trnL* and *ITS* matrices obtained in the TCS program (Clement et al., 2000). The size of the red rectangles and blue ellipses represent the haplotype frequency (**Table S1**).

A 13 base-pair (bp) inversion was found in the *trnL* ^(UAA)^ intron of cpDNA at the 1,209 position in the total matrix for *A. mapisaga* and *A. salmiana* subsp. *salmiana* “Ayoteco”. Therefore, in building our *trnL* haplotype network (**Figure 8A**), *A. mapisaga* and “Ayoteco” were grouped as haplotype H1. Using the *ITS* 1–4 marker, we only found a single base change (C–A) at position 602 in a 635-bp-long matrix, and polymorphisms were observed in *A. mapisaga*. Thus, the *ITS* haplotype network (**Figure 8B**) indicated a haplotype per species separated by a single mutation, grouping “Ayoteco” with *A. salmiana*. Finally, the combined matrix showed three haplotypes (**Figure 8C**): *A. mapisaga* (H1) separated by only one mutation from “Ayoteco” (H2) and the remaining *A. salmiana* taxa (H3). This latter haplotype is separated from the other two by various steps.

**Figure 9:**
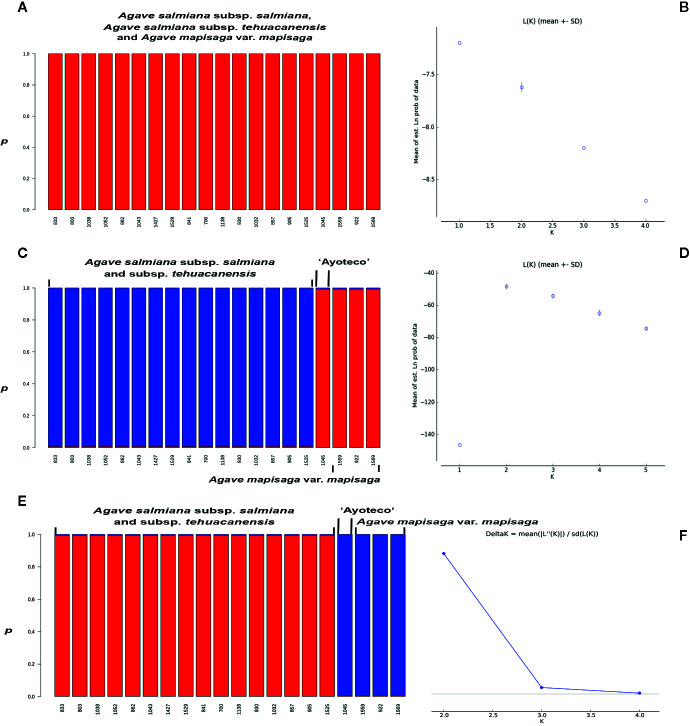
Individuals’ assignment analysis using the Bayesian statistic with the *p* co-ancestry coefficient in STRUCTURE and probability values for **(A**, **B)** *ITS* (K=1), **(C)** *trnL* (K=2), and **(D**, **E)** combined matrix (K=2); with the optimum genetic cluster number (K) based on Evanno’s ΔK statistic calculated in the STRUCTURE HARVESTER program **(F)**, except for *ITS*. The Evanno’s ΔK and probability values graphs for *trnL* are quite similar to the ones from the combined matrix, these were not presented.

In the STRUCTURE assignment analysis, we obtained a ΔK probability value supporting two clusters in *trnL* intron and combined matrices (**Figures 9C–F**). Each cluster or genetic pool corresponded with a species. Specifically, cluster 1 corresponded with *A. salmiana* subsp. *salmiana* and *A. salmiana* subsp. *tehuacanensis* and cluster 2 with *A. mapisaga* var. *mapisaga*. Only *A. salmiana* subsp. *salmian* “Ayoteco” was the exception and was shown to share the same genetic pool as *A. mapisaga* var. *mapisaga* (cluster 2). In the case of the *ITS* marker, only one cluster was identified with a K = 1 (**Figure 9A**). For this last marker, there was no ΔK obtained because there was no variance found between the different runs, and the highest probability value reached 1 (**Figure 9B**).

As previously mentioned, classical taxonomy based on morphological traits identifies “Ayoteco” as *A. salmiana* subsp. *salmiana*, even though this landrace shares traits with both species. However, the morphological analysis grouped “Ayoteco” and *A. mapisaga* var. *mapisaga*, even though they are not identical. Therefore, with respect to “Ayoteco,” the genetic data are not congruent, although the combined data analysis supports its close relationship with *A. mapisaga* var. *mapisaga*.

The history retrieved from each genome (either plastid or nuclear) tends to be different because of its origin, evolutionary processes, and manner of inheritance. Chloroplasts are haploid with maternal inheritance, almost nonexistent recombination, and a lower mutation rate compared to the nuclear genome, but higher than nuclear ribosomal DNA (Palmer et al., 1988; Wheeler et al., 2014; Ferradini et al., 2017). Many plant studies have easily found sufficient phylogenetic resolution from plastid genome markers (Yang et al., 2013; Wheeler et al., 2014).

There is a much discussion over whether the results of combined analyses are accurate since two different evolutionary histories are combined. However, these histories are not entirely independent because they have the same species history (Gadagkar et al., 2005; Degnan and Rosenberg, 2009). Some studies have found well-supported trees with high resolution from analyzing total combined data (Gadagkar et al., 2005; Degnan and Rosenberg, 2009). In the plastid genome, inversions are more rare than single nucleotide substitutions (Palmer et al., 1988); thus, in this case, the use of the *trnL* intron could be more plausible than the *ITS* marker. Ultimately, these approaches represent hypotheses that can be re-addressed in future studies to search for further phylogenetic resolution for the taxa in question, as the genetic data presented herein are not conclusive. It is necessary to explore a larger number of *Agave* species (paying special attention to the *Salmianae* group, Gentry, 1982) and a larger set of molecular markers to find ones that would be capable of providing a more complete and resolved phylogenetic history in order to better understand the genetic diversity of pulque-producing *Agave* species and landraces.

Currently, most studies on the *Agave* genus have been carried out at the population level using DNA fingerprint markers. Using microsatellite molecular markers (SSRs), Figueredo et al. (2015) found that *A. inaequides* (used for mezcal and pulque production) shows high genetic diversity and low structuring and that genetic diversity was mainly associated with the environment or the samples’ region of origin. This could imply that there is variability among populations of landraces or throughout their geographic distribution, which highlights the need to carry out further studies on their morphological and genetic diversity throughout their geographic distribution. Later, once again using microsatellite molecular markers (SSR), Figueredo et al. (2017) observed that the species used for mezcal and pulque production (*Agave inaequides*, *Agave hookeri*, and *Agave cupreata*) tended to form genetic pools corresponding with the species level, even though *A. hookeri* is mixed with cultivated *A. inaequides* plants. In the present study, *A. salmiana* and *A. mapisaga* could similarly represent separate genetic pools, with the exception of “Ayoteco” (**Figure 9**). Alvarez-Ríos et al. (2020) also found through SSR markers that *A. salmiana* and *A. mapisaga* have distinguishable genetic pools by species, with moderate genetic flow between these species. A cluster showing similarities to *Agave americana* was also found, possibly a hybrid of *A. salmiana* and *A. americana*. Microsatellites are especially useful for the study of interpopulational variation. In particular, chloroplast microsatellites (cpSSRs) are useful for testing hypothesis on the hybridization between species or populations (Wheeler et al., 2014). However, cpSSRs have been little used in studies on agave and could be one alternative for distinguishing the genetic pools of *A. salmiana* and *A. mapisaga* and possible introgression events.

Because of their low selection intensity, the landraces studied herein may be assigned to genetic clusters at the species level in contrast to extreme examples like *Agave tequilana* “Azul”, which is intensively and continuously cloned to produce tequila. *A. tequilana* “Azul” is recognized as having an identity distinct from other possibly closely related species (Trejo et al., 2018). With respect to pulque agaves, there is a possibility that morphological vegetative traits are plastic and reflect growers’ knowledge of different landraces and the common names given to different forms. Accordingly, such plasticity may be environmental and directed by the selection of plants or, occasionally, may result from genetic flow between them when plants eventually flower following abandonment or growers forget to harvest the aguamiel.

## Conclusions

The perception of the diversity of pulque agaves is high among growers, as reflected by the high number of common names. However, most morphological studies have only analyzed vegetative characters and have only been able to differentiate landraces at the species level. The sequencing analyses carried out herein enabled genetic variants generally associated with genetic pools at the species level to be identified. However, the landraces from the pulque production agaves could be different from each other, since they have origin in different wild plants and have different uses. Furthermore, crop abandonment allows for genetic flow events. In contrast, the intensive production of certain landraces through restrictive asexual reproduction or the complete abandonment of certain landraces could lead to their eventual disappearance.

The identity of *A. salmiana* subsp. *salmiana* “Ayoteco” is still not clear, this might be a hybrid between *A. salmiana* and *A. mapisaga* or we just might be observing gene flow between “Ayoteco” and *A. mapisaga*. In order to acquire further understanding about pulque landraces’ taxonomic identity, it is necessary to include more characters in the analysis, including reproductive and biochemical characters, as well as polymorphisms in a single nucleotide (SNP). It is also recommendable to perform population analyses of *Agave* landraces throughout their geographic distribution and compare these to their wild relatives.

In Tlaxcala, the conservation of *Agave* landraces with a local distribution can be strengthened through their taxonomic identification and continued local use. However, the conservation of their vast richness also depends on avoiding their replacement with other landraces that could lead to their extinction, such as in the Yucatan Peninsula, where local landraces (henequen) have been displaced, or in Jalisco, where traditional varieties once used for tequila production have been substituted by the blue agave.

## Data Availability Statement

The datasets generated for this study can be found in the GenBank (see **Table S2**).

## Author Contributions

LT participated in collecting, taking morphological characters measurements, obtaining sequences, analyzing kin relationships, writing and editing the article. MR and DC-T did the statistical analysis of morphological characters, and participated in writing and editing the article. ER-G took part in collecting, taking morphological characters measurements and making some of the figures. LM-C also took part in collecting and measuring morphological characters.

## Conflict of Interest

The authors declare that the research was conducted in the absence of any commercial or financial relationships that could be construed as a potential conflict of interest.
