# Supplementary material for: Morphological Diversity and Genetic Relationships in Pulque Production Agaves in Tlaxcala, Mexico, by Means of Unsupervised Learning and Gene Sequencing Analysis
Source: Front Plant Sci. 2020 Sep 8;11:524812. doi: 10.3389/fpls.2020.524812 (PMC7505951; doi:10.3389/fpls.2020.524812)
Supplement: Supplementary file 1 [file DataSheet_1.docx]

Supplementary Material

Supplementary Data 1

*Agave salmiana* Otto ex Salm-Dyck subsp. *salmiana*

Botanical description. Distinguished by massive and dense rosettes. Fleshy lanceolate to widely lanceolate leaves 1.5 to 2.2 m long and 20 to 35 cm wide, erect or recurved. Leaf margins straight or undulated, ending in sigmoid shaped apex, elongated and acuminate with a long terminal spine robust to subulate. Leaf margin 0.5 to 1 cm long and teeth 1 to 2 cm wide with a serrated configuration. Plant coloration dark green or light green to yellowish green. Robust inflorescence 6 to 8 m long, generally pyramidal in shape; longest ramifications reaching up to 1 m in length. Inflorescence formed by 15 to 25 umbels. Peduncle covered by fleshy bracts; tepals of yellow coloration and light green tissue covering the ovary.

The subspecies name applies to both cultivated and naturalized plants (García-Mendoza, 2011). No botanical description is available for the landrace. Descriptions of landraces reflect observations in their habitat and average values for some morphological characters measured herein.

*Agave salmiana* subsp. *salmiana* ‘Amarillo’

Rosettes with an average size of 198 cm and a diameter of 253 cm. Leaves green-yellow with an average length of 149 cm. Only a few individuals were found dispersed in the San Pedro cornfields, Tlacuapan, Chiautempan, Tlaxcala. In the past, this landrace was utilized to obtain fiber for *cuartas* (a type of rope used in carnivals) production; now, aguamiel is also harvested from it. It is not known whether it is native to the locality or not (Fig. 2A, Table 1).

*Agave salmiana* subsp. *salmiana* ‘Ayoteco’

One of the largest agaves studied herein with an average height of 282 cm and average diameter of 414 cm. Leaves dark blue-green colored, often speckled with black spots; some leaves flaccid with a length of up to 251 cm. Terminal spine short of 49 mm and curved. This cultivar was only found in Nanacamilpa municipality, where it is cultivated for aguamiel, mixiote (leaf cuticles used for cooking meat), and leaves for lining roast pits. Some local inhabitants recognize the plant as native, while others indicate it was introduced. There are no known wild relatives of this cultivar (Gentry, 1982; Fig. 2B, Table 1).

*Agave* *salmiana* subsp. *salmiana* ‘Colorado’

Average height and diameter of 210 cm and 261 cm, respectively. Leaves with an average length of 155 cm, yellow-green in color, and reddish edges. Leaves tend to dry out toward the apices. Specimens were observed in Nanacamilpa, Atlangatepec, and El Camen Tequexquitla. Despite producing small amounts of aguamiel compared to other landraces, a small population of this cultivar is grown due to the sweet taste of its sap. Local inhabitants recognize the plant as native (Fig. 2C, Table 1).

*Agave* *salmiana* subsp. *salmiana* ‘Chalqueño’

One of the largest agaves studied herein, reaching heights of around 304 cm and a diameter of 422 cm. Grows vertically upwards with 247-cm-long leaves, green with slightly grayish hues. It was only found in Nanacamilpa, where it is cultivated for its aguamiel. Its place of origin is unknown (Fig. 2D, Table 1).

*Agave salmiana* subsp. *salmiana* ‘Chino’

This agave gets its name *chino* (curly) from its recurved leaf margins and abundant teeth. Green coloration. Average height of 222 cm, with a diameter of 346 cm and a leaf length of 173 cm. It was observed only in Alpotzonga and Ixtlacuixtla, where it is cultivated to obtain aguamiel and leaves for lining roast pits. Its place of origin is unknown (Fig, 2E; Table 1).

*Agave salmiana* subsp. *salmiana* ‘Manso’ and ‘Manso Listado’

This is the most intensely exploited and widely distributed cultivar in Tlaxcala. Medium sized, with an average height of 258 cm and average diameter of 380 cm. Leaves flexible and green, with a straight terminal spine. Some differences in size can be observed: The largest individuals were observed in Tlaxco and Atlangatepec, while smaller ones were seen in Nanacamilpa. It has many uses, including for its aguamiel, mixiote, and leaves (Fig. 2F, Table 1). A plant named ‘Manso Listado’, with yellow-striped leaves, was also located. The people of Tlaxcala recognize these varieties as native plants.

*Agave salmiana* subsp. *salmiana* ‘Matecón’

A few plants were observed between cornfields in Chiautempan and Teacalco, both inside the fields and as living fences. Similar size and color to ‘Manso’ agaves, but leaves are shorter, concave, and spoon-like. Local inhabitants recognize it as a native plant (Fig. 2G, Table 1).

*Agave salmiana* subsp. *salmiana* ‘Prieto’

Widely distributed in Tlaxcala, this cultivar can also be observed growing naturally on hills in the region (‘Prieto Silvestre’ and ‘Prieto Listado’). Average height of 202 cm and average diameter of 258 cm. Leaves with very dark green hues. It is cultivated for aguamiel in Atlangatepec, Chiautempan, and Santa Cruz, Tlaxcala (Fig. 2H, Table 1). Wild populations were observed in Atlangatepec, Teacalco, and El Carmen Tequexquitla.

*Agave salmiana* subsp. *salmiana* ‘Púa Larga’

Similar in size to ‘Manso’ but with a higher number of leaves (average values not available) and a terminal spine reaching up to 13 cm in length. Locals indicate that eight-year-old plants can be used to produce aguamiel, and the cuticle is easily broken when pulled. It was only observed in Nanacamilpa. Inhabitants state that it is not a native plant and has been introduced in the last few decades, but its origin is unknown (Fig. 2I, Table 1).

*Agave salmiana* subsp. *salmiana* ‘Wexometl’

Medium-sized plants similar to the ‘Manso’ cultivar. Lanceolate to broadly lanceolate leaves with a dark green coloration, not convex, with an obtuse apex. Only a few individuals were found in Españita.

*Agave salmiana* subsp. *salmiana* ‘Xilomelt’

Average height of 222 cm and average diameter of 319 cm. Large-sized agave that does not tilt over completely. Yellowish-green leaves 178 cm in length. Locals appreciate the flavor and great quantities of aguamiel produced by this agave. Only a few individuals were observed in Españita and Nanacamilpa (Fig. 2J, Table 1).

*Agave salmiana* subsp. *tehuacanensis* ‘Tepezorra’

Wild subspecies with a formal botanical description. Small rosettes, acaulescent, with an urceolate shape. Both height and diameter of 1 to 1.5 m. Low number of leaves compared to other landraces, with a length of 50 to 70 cm and width of 15 to 20 cm, widely lanceolate, erect to recurved, concave in the middle, and light green in color. Leaf apex acuminate and short; crenate margin also found, strongly armed with mammillated prominences toward the middle part of the leaf. Margins with teeth 1.0 to 1.5 cm long and 1.0 to 2.0 cm wide. Apex ends in a strong spine, widely fluted on the adaxial surface.

Short inflorescence 3.5 to 5 m in height with 10 to 15 umbels; primary branches 40 to 50 cm long. Peduncle covered with coriaceous bracts (García-Mendoza, 2011). A wild plant was observed in a ravine near El Carmen Tequexquitla. It is not commonly used for aguamiel extraction (Fig. 2K, Table 1).

*Agave mapisaga* Trel. var. *mapisaga*

Plants with large and dense rosettes. Leaves linear, fleshy, rigid to flexible, with a concave shape toward the middle part. Apex elongate with acuminate ending in a short, robust, fluted spine. Margin with small, straight, closely spaced teeth. Distinguished by its green to glaucous green coloration. Massive inflorescence over 8 m in height. Floral peduncle covered in large fleshy bracts. Flowers large and fleshy; tepals often reddish in bud but yellow when open (Gentry, 1982).

Studied populations had an average height of 261 cm, average diameter of 397 cm, and average leaf length of 216 cm (Fig. 2L, Table 1). It is known by several common names such as ‘Penca Larga’, ‘Palmilla,’ and ‘Savililla,’ which all identify the same ‘cultivar.’ Few crop fields were observed (Atlangatepec, Españita). Plants were often found as scattered individuals in fields or as ornamental plants. It is utilized to obtain aguamiel. Its place of origin remains unknown.

Supplementary Tables

Table S1. Voucher information (all at MEXU), haplotype number, and GenBank accession numbers of the samples sequenced; the localities corresponds to a municipality in Tlaxcala, except *Agave angustifolia*, which corresponds to the state of Jalisco.

| Species | ‘Landrace’/Wild plant | Collection no. | Municipality | Haplotype_cp_^a^ | Haplotype_n_^b^ | Haplotype_cp+n_^c^ | *trnL* | ITS |
| --- | --- | --- | --- | --- | --- | --- | --- | --- |
| *A. salmiana* subsp. *salmiana* | ‘Amarillo’ | 833 | Chiautempan | 2 | 2 | 3 | MN56042 | MN056950 |
| *A. salmiana* subsp. *salmiana* | ‘Ayoteco’ | 1045 | Nanacamilpa | 1 | 2 | 2 | MN56043 | MN056951 |
| *A. salmiana* subsp. *salmiana* | ‘Ayoteco’ | 1559 | Nanacamilpa | 1 | 2 | 2 | MN56044 | MN056952 |
| *A. salmiana* subsp. *salmiana* | ‘Colorado’ | 803 | Atlangatepec | 2 | 2 | 3 | MN56045 | MN056953 |
| *A. salmiana* subsp. *salmiana* | ‘Chalqueño’ | 1038 | Nanacamilpa | 2 | 2 | 3 | MN56046 | MN056954 |
| *A. salmiana* subsp. *salmiana* | ‘Chino’ | 1052 | Ixtlacuixtla | 2 | 2 | 3 | MN56047 | MN056955 |
| *A. salmiana* subsp. *salmiana* | ‘Manso’ | 862 | Españita | 2 | 2 | 3 | MN56048 | MN056956 |
| *A. salmiana* subsp. *salmiana* | ‘Manso’ | 1043 | Nanacamilpa | 2 | 2 | 3 | MN56049 | MN056957 |
| *A. salmiana* subsp. *salmiana* | ‘Manso’ | 1427 | Tlaxco | 2 | 2 | 3 | MN56050 | MN056958 |
| *A. salmiana* subsp. *salmiana* | ‘Manso Listado’ | 1529 | Nanacamilpa | 2 | 2 | 3 | MN56051 | MN056959 |
| *A. salmiana* subsp. *salmiana* | ‘Matecón’ | 841 | Chiautempan | 2 | 2 | 3 | MN56052 | MN056960 |
| *A. salmiana* subsp. *salmiana* | ‘Prieto’ | 700 | Chiautempan | 2 | 2 | 3 | MN56053 | MN056961 |
| *A. salmiana* subsp. *salmiana* | Prieto Listado | 1138 | El Carmen Tequexquitla | 2 | 2 | 3 | MN56054 | MN056962 |
| *A. salmiana* subsp. *salmiana* | Prieto Silvestre | 930 | Teacalco | 2 | 2 | 3 | MN56055 | MN056963 |
| *A. salmiana* subsp. *salmiana* | ‘Púa Larga’ | 1032 | Nanacamilpa | 2 | 2 | 3 | MN56056 | MN056964 |
| *A. salmiana* subsp. *salmiana* | ‘Xilomelt’ | 857 | Españita | 2 | 2 | 3 | MN56057 | MN056965 |
| *A. salmiana* subsp. *salmiana* | ‘Wexomelt’ | 905 | Españita | 2 | 2 | 3 | MN56058 | MN056966 |
| *A. salmiana* subsp. *tehuacanensis* | Tepezorra | 1525 | El Carmen Tequexquitla | 2 | 2 | 3 | MN56059 | MN056967 |
| *Agave mapisaga* var. *mapisaga* | ‘Palmilla’ | 922 | Españita | 1 | 1 | 1 | MN56060 | MN056968 |
| *Agave mapisaga*  var. *mapisaga* | ‘Penca Larga’ | 1569 | Tlaxcala | 1 | 1 | 1 | MN56061 | MN056969 |

^c^Combined plastid + nuclear data haplotypes (Haplotype_cp+n_) numbers correspond to those in Fig. 8.

Table S2. Tested primers for interspecific variation between *Agave salmiana* subsp. *salmiana*, *Agave salmiana* subsp. *tehuacanensis* and *Agave mapisaga* var *mapisaga*.

| Name | Code | Non-coding region | Genome | Reference | Results |
| --- | --- | --- | --- | --- | --- |
| *trnH* ^(GUG)^-*psbA* | *trnH-psbA* | Intergenic spacer | Chloroplast | Hamilton, 1999 | No variation |
| *trnS* ^(GCU)^-*trnG* ^(UCC)^ | *trnS-trnG* | Intergenic spacer | Chloroplast | Hamilton, 1999 | No variation |
| *trnT* ^(UGU)^-*trnL* ^(UAA)^5'exon | *TabA*(a)-*TabB*(b) | Intergenic spacer | Chloroplast | Taberlet et al., 1991 | Did not amplify |
| *trnL* ^(UAA)^5'exon-*trnL* ^(UAA)^3'exon | *TabC*(c)-*TabD*(d) | Intron | Chloroplast | Taberlet et al., 1991 | Variation found |
| *trnL* ^(UAA)^3'exon-*trnF* ^(GAA)^ | *TabE*(e)-*TabF*(f) | Intergenic spacer | Chloroplast | Taberlet et al., 1991 | No variation |
| *trnQ* ^(UUG)^-*5´rps16* | *trnQ*-*rps16* | Intergenic spacer | Chloroplast | Shaw et al., 2007 | Multiple bands |
| *3´rps16-5´trnK* ^(UUU)^ | *rps16-trnK* | Intergenic spacer | Chloroplast | Shaw et al., 2007 | No variation |
| *psbJ-petA* | *psbJ-petA* | Intergenic spacer | Chloroplast | Shaw et al., 2007 | Did not amplify |
| *rpl*32*- trnL* *(UAG)* | *rpl32-trnL* | Intergenic spacer | Chloroplast | Shaw et al., 2007 | * |
| *ITS*1*-ITS*4 | *ITS* (1-4) | Internal transcribed spacer | Nuclear ribosomal | Bayer et al., 1996 | Variation found |

* This region is identical to a fragment in *TrnL*/^(UAA)^ intron (Hamilton, 1999) therefore it was not used.

Table S3. Useful characters for the visualization of morphological clusters in agaves utilized in the production of mezcal and pulque.

| RH | RD | LL | LWM | LTB | LN | LC | TN | TL | TW | DT | TSL | DTS | CT | LFW | LDW | pH | °Brix | SSS | IL | SBL | PL | ETL | AL | CL | SL | PMS | Referencia |
| --- | --- | --- | --- | --- | --- | --- | --- | --- | --- | --- | --- | --- | --- | --- | --- | --- | --- | --- | --- | --- | --- | --- | --- | --- | --- | --- | --- |
|  |  |  |  |  |  |  | **X** |  |  |  | **X** |  |  |  |  |  |  |  |  |  |  |  |  |  |  |  | Alfaro-Rojas et al., 2007 |
| **X** | **X** | **X** |  | **X** |  |  | **X** | **X** | **X** | **X** |  |  | **X** |  |  |  |  |  |  |  |  |  |  |  |  |  | Mora-López et al., 2011 |
| **X** |  |  | **X** | **X** |  | **X** |  |  |  |  |  |  |  | **X** | **X** | **X** | **X** | **X** |  |  |  |  |  |  |  |  | Rocillo, 2015 |
| **X** |  | **X** | **X** |  |  | **X** | **X** | **X** |  | **X** | **X** |  |  |  |  |  |  |  |  |  |  |  |  |  |  |  | Vargas-Ponce et al., 2007 |
| **X** | **X** |  | **X** |  |  |  | **X** | **X** | **X** | **X** |  | **X** |  |  |  |  |  |  |  |  |  |  |  |  |  |  | Rodríguez-Garay et al., 2009 |
| **X** | **X** | **X** | **X** |  | **X** |  |  |  |  |  |  |  |  |  |  |  |  |  |  |  |  |  |  |  |  |  | Torres-Moran et al., 2013 |
| **X** | **X** | **X** | **X** |  |  |  | **X** | **X** | **X** | **X** | **X** |  |  |  |  |  |  |  |  |  |  |  |  |  |  |  | Figueredo et al., 2014 |
| **X** |  |  | **X** | **X** |  | **X** |  |  |  |  |  |  |  |  |  |  |  |  |  | **X** |  |  | **X** | **X** | **X** |  | Vázquez-Pérez, 2015 |
| **X** | **X** | **X** | **X** |  |  |  | **X** | **X** | **X** | **X** | **X** |  |  |  |  |  |  |  |  |  | **X** | **X** | **X** | **X** | **X** |  | Figueredo et al., 2017 |
|  |  | **X** |  |  | **X** |  | **X** | **X** |  |  | **X** |  |  |  |  |  |  |  | **X** | **X** | **X** | **X** | **X** |  | **X** | **X** | Rivera-Lugo et al., 2018 |
| **X** | **X** | **X** | **X** |  |  |  | **X** | **X** | **X** | **X** | **X** | **X** |  |  |  |  |  |  |  |  |  |  |  |  |  |  | Álvarez-Ríos et al., 2020 |
| **X** | **X** | **X** | **X** | **X** | **X** |  | **X** | **X** | **X** | **X** | **X** | **X** | **X** |  |  |  |  |  |  |  |  |  |  |  |  |  | In this work |

RH=Rosette, height; RD=Rosette, diameter; LL= Leaf, length; LWM=Leaf, width at mid length; LTB=Leaf, thickness at base; LN=Leaf number; LC=Leaf, color; TN=Teeth number; TL=Tooth, length; TW=Tooth, width; DT=Distance between teeth; TSL=Terminal spine, length; DTS=Distance between last tooth and terminal spine; CT=Cuticle, thickness; LFW=Leaf, fresh weight; LDW=Leaf, dry weight; SSS=Short broad stem, total sugars; IL=Inflorescence, length; SBL=Sterile bract, length; PL=Pedicel, length; ETL=External tepal, length; AL=Anther, length; CL=Capsule, length; SL=Seed, length; PMS=Proportion of mature seeds. The red marks indicate the characters that were significant in more than 50% of the reviewed works.

Table S4. Comparison of studies of morphological characters of agave landrace utilized in the production of pulque and mezcal.

| Species | Use | Landrace | Number of  individuals | Number of characters | Forms group with | Reference |
| --- | --- | --- | --- | --- | --- | --- |
| *Agave salmiana*  *Agave salmiana*  *Agave mapisaga* | pulque | ‘Manso’  ‘Ayoteco’  ‘Carrizo’  ‘Verde’  ‘Negro’  ‘Xilomelt’ | 25  25  25  25  25  25 | 6 | Group 1: ‘Carrizo’.  Group 2: ‘Negro’.  Group 3: ‘Xilomelt’  Group 4: ‘Manso’, ‘Ayoteco’ y ‘Verde’ | Alfaro-Rojas et al., 2007 |
| *Agave macroculmis*  *Agave mapisaga*  *Agave mapisaga* var. *lisa*  *A. salmiana* ssp. *crassispina*  *A. salmiana* var. *ferox*  *Agave salmiana var. salmiana*  *Agave tecta* | pulque | ‘Serrano’  12  ‘Morado’  13  3  22  ‘Grande’ | 6  6  6  6  6  6  6 | 48 | In a relative manner. I. *Agave mapisaga*. II.  *Agave salmiana* subsp. *salmiana*. III. *A. salmiana* ssp. *crassispina* y *A. salmiana* var. *ferox* | Mora-López et al., 2011 |
| *Agave inaequidens*  *Agave hookeri* | pulque, mezcal  pulque | ‘Maguey Alto’  ‘Maguey Manso’ | ¿?  ¿¿ | 25  25 | In *Agave inaequidens*, can be distinguish between wild and landrace.  They can tell apart *A. inaequidens* from *A. hookeri*, even though some cultivated individuals may hybridize. | Figueredo et al., 2014 |
| *Agave salmiana*  Agave mapisaga  *Agave sp.* | pulque | ‘Manso’  ‘Cenizo’  ‘Negro’ | 15  15  15 | 35 | Greater similarity between ‘Manso’ and ‘Negro’ | Rocillo, 2015 |
| *Agave inaequidens*  *Agave hookeri*  *Agave cupreata* | pulque, mezcal  pulque  mezcal | ‘Maguey Alto’  ‘Maguey Manso’  ‘Papalote’ | ¿?  ¿?  ¿? | 39  39  39 | Species differentiation. Differentiation between wild and cultivated *A. inaequidens*. Similarities between cultivated *A. inaequidens* and *A. hookeri*. | Figueredo et al., 2017 |
| *Agave salmiana*  *Agave mapisaga* var. *mapisaga*  *Agave americana?* | pulque | ‘Verde, ‘Negro’  Tarímbaro’, ‘Listoncillo’  ‘Blanco’, ‘Carrizaleño’, ‘Cenizo’ | 17, 18  2, 27  2, 4, 2 | 20 | Species differentiation. | Álvarez-Ríos et al., 2020 |
| *A. angustifolia*  *A. rhodacantha*  *A. guadalajarana* | mezcal | 16  ‘Ixtlero Amarillo’  ‘Garabato’ | 4-10  8-9  10 | 19 | Group I: Ixtlero Amarillo y Cimarrón de Zapotitlán.  Group II: 15 landraces.  Group III: wild populations, mezcal Piña y Perempitz.  Group IV: ‘Agave Azul’.  Group V: ‘Prieto’,  ‘Presa Grande’. | Vargas-Ponce et al., 2007 |
| *Agave tequilana*  *Agave angustifolia* | mezcal | ‘Azul’  ‘Lineño’ | 17  16 | 13 | Azul forms a group close to ‘Lineño’ | Rodríguez-Garay et al., 2009 |
| *Agave tequilana*  Agave americana  *Agave maximiliana*  *Agave salmiana*  *Agave angustifolia* | mezcal | ‘Azul’ | 100  10  10  10  5 | 6 | Group 1: *A. tequilana* y *A. angustifolia.*  Group 2: el resto. | Torres-Moran et al., 2013 |
| *Agave karwinskii* | mezcal | ‘Cachitún’  ‘Sierrudo’  ‘Cirial’  ‘Marteño’  ‘Tripón’  ‘Espina Negra’  ‘Madrecuishe’  ‘Bicuishe’  ‘Tobasiche’  ‘San Martín’  ‘Cuishe’ | 7  7  7  7  7  7  7  7  7  7  7 | 51 | Group A: ‘Tripón’, ‘Marteño’, ‘Tobasiche’, ‘San Martín’, ‘Madrecuishe’, ‘Bicuishe’, ‘Sierrudo’.  Group B: ‘Espina Negra’, ‘Cirial’, ‘Cuishe’, ‘Sierrudo’, ‘Cachitún’. | Vázquez-Pérez, 2015 |
| *Agave angustifolia rubescens*  *Agave angustifolia*  *Agave angustifolia*  *Agave rhodacantha*  *Agave tequilana* | mezcal | Wild  ‘Espadín’  Sonora  Oaxaca  ‘Azul’ | 5  5  5  5  5  5 | 46 | (*A. angustifolia* Oaxaca, *A. angustifolia* var. *rubescens*); (*A. angustifolia* Sonora (*A. tequilana* (*A. rhodacantha*, *A. angustifolia* Espadín))) | Rivera-Lugo et al., 2018 |

Supplementary Figures

**Figure S1.** Biplot showing information on both samples and variables.


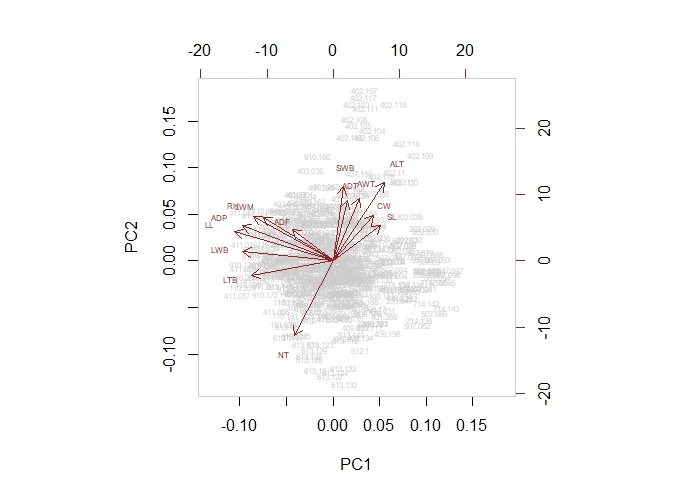


Supplementary References

Hamilton, M.B. (1999). Tour primer for the amplification of chloroplast intergenic regions with intraspecific variation. Mol. Ecol. 8, 513-525.

Rivera-Lugo, M., García-Mendoza, A., Simpson, J., Solano, E., Gil-Vega, K. (2018). Taxonomic implications of the morphological and genetic variation of cultivated and domesticated populations of the *Agave angustifolia* complex (Agavoideae, Asparagaceae) in Oaxaca, Mexico. Plant. Syst. Evol. 304, 969-979. <https://doi.org/10.1007/s00606-018-1525-0>.

Rocillo, Z, I. (2015). Caracterización morfológica y bioquímica de tres especies de maguey pulquero (*Agave* spp.) en la región de San Martín de las Pirámides, Estado de México. Dissertation, Universidad Autónoma Chapingo.

Rodríguez-Garay, B., Lomelí-Sención, J.A., Tapia-Campos, E., Gutiérrez-Mora, A. García-Galindo, J., Rodríguez-Domínguez, et al. (2009). Morphological and molecular diversity of *Agave tequilana* Weber var. *Azul* and *Agave angustifolia* Haw. var. *Lineño*. Ind. Crops. Prod. 29, 220-228. <https://doi.org/10.1016/j.indcrop.2008.05.007>.

Shaw, J., Lickey, E.B., Schilling, E.E., Small, R.L. (2007). Comparison of whole chloroplast genome sequences to choose noncoding regions for phylogenetic studies in angiosperms: the tortoise and the hare III. *American Journal of Botany* 94, 275-288.

Torres-Morán, M.I. Velasco-Ramírez, A.P., Hurtado-de la Peña, S.A., Rodríguez-García, A., Mena-Munguía, S. (2013). Variability and genetic structure in a comercial field of tequila plants, Agave tequilana Weber (AGAVACEA). Am. J. Agric. Biol. Sci. 8, 44-53. <https://doi.org/10.3844/ajabssp.2013.44.53>.

Vázquez-Pérez, N. (2015). Variación morfológica y genética *Agave kerwinskii* (Agavaceae) en los estados de Oaxaca y Puebla. Dissertation, Universidad Nacional Autónoma de México, México.
